# Mr. Abraham on the Influence of Civilization

**Published:** 1826-07

**Authors:** 


### INFLUENCE OF CIVILIZATION ON THE INCREASE AND PERPETUATION OF DISEASES.

In the last Number of our junior cotemporary of the North, there is a short paper on this subject, by Mr. Abraham, of Carlisle, in which we think there are many just and judicious reflections. That some of his conclusions may be carried a little too far, we do not deny; but we are convinced that his principles are correct in general. The increase of insanity and of other diseases in civilized life is the first subject touched on by Mr. Abraham, and here it is that he indulges in some curious speculations. The reason why we see so few instances of insanity, and indeed of chronic diseases, among the *uncivilized* tribes, is, he thinks, that the difficulty of procuring the necessaries of life, and their precarious supply, rapidly cuts short the existence of those whose physical or moral defects preclude their taking an equal share in the toils of the community, where, among certain tribes, parents even abandon such of their offspring as are incapacitated by congenital deformity for labour or warfare.

"But it is totally different in civilized life. Here the idiotic may prolong their existence, and perpetuate their species; for the degree of imbecility must be great indeed, to disable an individual from expending a stated income, or from partaking in the peaceful and monotonous labours of agriculture and manufactures; they are not left to perish, helpless and unfriended, as in the wilds of America; or even, if we may believe some travellers, as in the north of Europe and Asia. The madman who, in the one situation, would quickly fall a victim to his unconscious crimes and eccentricities, is in the other protected by equitable laws; has his property guarded by official trustees; all the resources of art are expended in mitigating his calamity, and he but too often leaves behind him a numerous progeny, who feel through all their ramifications, the evil entailed on them by their ancestor, and extend it in every direction. Blindness, deafness, and dumbness, which, in the savage state, are incompatible with those exertions by which alone life can be supported, we too often see perpetuated in Europe, in one family, from generation to generation. Individuals with tuberculated lungs, who, in the vicissitudes of savage life, would quickly fall victims to pulmonary inflammation, are protected by the refinements of civilization from the inclemency of the seasons; and are usually preserved, by medical interference, and the comforts of domestic life, long enough to leave families to lament their untimely loss. What multitudes of people do we not daily see, who, by judicious diet, change of air, medical treatment, surgical operations, and other remedial powers, unknown to the uncultivated savage, are rescued from the mortal grasp of pleuritic, of gouty, of rheumatic, or of strumous inflammation, to drag about for the remainder of life a broken constitution, or a mutilated person, and to transmit to posterity a precarious existence, and the seeds of irremediable disease!" 248.

Mr. Abraham thinks that the introduction of inoculation, and still more of vaccination, has increased the list of chronic or constitutional maladies; and we think it not improbable. Many infirm constitutions —thousands of children with the germs of hereditary disease, would be annually cut off by the violence of the natural small pox, but who resist the milder form of inoculation, and who, of course, suffer nothing from vaccination. But however probable this may appear to the medical philosopher, we do not see how it can be acted on by the statesman or legislator.

Speaking of mental and corporeal diseases, our author makes the following eloquent reflections.

"Perhaps, however, we may rationally indulge a hope, that, in the hidden resources of a bountiful Providence, there is some remedy in store to check the progress of this terrible evil; and that beautiful system of compensation, which we observe in the government of the human race, and which seems to leave no evil, whether moral or physical, without its appropriate remedy, encourages the idea. This check, if any such be found, must, I am convinced, be looked for in the moral feelings of mankind; for when the formidable nature of the evil becomes more fully appreciated, individuals of sane families will become loth to unite themselves to those who are predisposed to mental or bodily disease, and thus the families of the latter description, may possibly become insulated and gradually worn out, from celibacy, and the concentration of infirmity from mutual alliances. And, unless the operation of the causes I have mentioned, as tending in civilized society to the increase of hereditary morbific predisposition, be corrected in this, or some similar manner, a few more centuries of civilisation, refinement, and luxury, will, in all probability, find the human race one melancholy accumulation of fatuity, madness, and disease." 333.

We do not take quite so gloomy a view of the case as is represented in the latter part of the foregoing passage. There are many sources of health springing up in modern times, and it is quite certain that the average range of life is now extended beyond what it was fifty or sixty years ago—that is, that the probability of living a certain number of years, from any given period, is now greater than it was, as the various Life Assurance Societies well know. This is probably owing to temperance, increased comforts in living, and the extension of more correct medical knowledge.

